# Supplementary material for: Genomic prediction of growth in a commercially, recreationally, and culturally important marine resource, the Australian snapper (Chrysophrys auratus)
Source: G3 (Bethesda). 2022 Jan 20;12(3):jkac015. doi: 10.1093/g3journal/jkac015 (PMC8896003; doi:10.1093/g3journal/jkac015)
Supplement: jkac015_Supplementary_Data [file jkac015_supplementary_data.docx]

**Supplementary File**

**Table S1** Filtering steps and number of SNPs retained for the two groups (small and large) of Australian snapper (*Chrysophrys auratus*).

| **Filtering step** | **SNPs** |
| --- | --- |
| Raw SNP catalogue | 1,273,017 |
| Genotyped in >80% individuals, minor allele >3%, bi-allelic, allele Balance >20% and <80% | 38,368 |
| Remove indel | 32,025 |
| Read quality (ratio quality/coverage depth >0.2) mapping quality (Q≥30) | 29,425 |
| Read depth (>20 and ≤ mean depth + 2 * standard deviation) | 28,223 |
| Hardy-Weinberg equilibrium (p < 0.05) | 21,436 |
| Calling rate (Mismatch genotypes in ≥2 replicate) | 19,700 |
| Missing data in each size group <1% | 17,490 |
| Linkage disequilibrium (remove SNPs within 100 Kb and R^2^ ≥ 0.5) | 11,588 |

**Table S2** Chromosome size, SNPs per chromosome (bp = base pairs), and SNP-based heritability (h^2^_SNP_) per chromosome for Australian snapper (*Chrysophrys auratus*)

| **Chr** | **bp** | **#SNP** | **SNP/100Kbp** | **h^2^_SNP_** |
| --- | --- | --- | --- | --- |
| LG1 | 35,796,517 | 697 | 1.95 | 11.18 |
| LG2 | 36,161,541 | 835 | 2.31 | 4.38 |
| LG3 | 34,661,116 | 849 | 2.45 | 0.49 |
| LG4 | 33,120,650 | 934 | 2.82 | 1.58 |
| LG5 | 35,422,278 | 728 | 2.06 | 2.50 |
| LG6 | 28,599,003 | 739 | 2.58 | 3.61 |
| LG7 | 34,240,341 | 725 | 2.12 | 0.66 |
| LG8 | 37,046,140 | 816 | 2.20 | 0.52 |
| LG9 | 27,277,866 | 739 | 2.71 | 0.66 |
| LG10 | 33,112,362 | 737 | 2.23 | 0.71 |
| LG11 | 38,628,475 | 710 | 1.84 | 4.41 |
| LG12 | 26,212,225 | 644 | 2.46 | 1.27 |
| LG13 | 33,878,147 | 689 | 2.03 | 2.76 |
| LG14 | 29,913,436 | 699 | 2.34 | 0.90 |
| LG15 | 28,243,467 | 723 | 2.56 | 0.70 |
| LG16 | 30,315,390 | 743 | 2.45 | 16.43 |
| LG17 | 24,333,991 | 665 | 2.73 | 0.98 |
| LG18 | 32,765,680 | 677 | 2.07 | 5.43 |
| LG19 | 28,261,070 | 634 | 2.24 | 0.77 |
| LG20 | 26,584,312 | 542 | 2.04 | 0.30 |
| LG21 | 28,350,763 | 729 | 2.57 | 0.64 |
| LG22 | 29,453,248 | 622 | 2.11 | 8.89 |
| LG23 | 20,385,525 | 547 | 2.68 | 1.54 |
| LG24 | 17,221,635 | 436 | 2.53 | 0.95 |
| LG25 | 3,820,059 | 40 | 1.05 | 0.03 |

**Table S3** Model summary of bayesR results on the genomic architecture of growth of Australian snapper (*Chrysophrys auratus*). NSNP = number of SNPs use in the model to explain the variation, Vg = Genetic variance explained by SNPs in the model, Ve = residual variance, Nk[1-4] = number of SNPs in mixture component 1-4, Vk[1-4] = SNP effects in mixture component 1-4. Bayesian model assumes a variance in each mixture component of 1 = 0, 2 =0.0001, 3=0.001, 4=0.01. SNP-based heritability (h^2^_SNP_) explained by the whole model and each of its mixture components.

|  |  | **h^2^_SNP_** |
| --- | --- | --- |
| **Nsnp** | 2,102 |  |
| **Va** | 0.2102 | 75.73 |
| **Ve** | 0.0674 |  |
| **Nk1** | 15,388 |  |
| **Nk2** | 1,813 |  |
| **Nk3** | 229 |  |
| **Nk4** | 60 |  |
| **Vk1** | 0.0000 | 0 |
| **Vk2** | 0.0386 | 13.90 |
| **Vk3** | 0.0487 | 17.55 |
| **Vk4** | 0.1221 | 44.01 |

**Table S4** Annotation results of 65 contigs of 10kbp containing the 100 SNPs candidates for growth in Australian snapper (*Chrysophrys auratus*).

| **Genomic region** | **SNP in Exon** | **UniProt^1^ Accession number** | **Protein name** | **Gene symbol** | **Species** | **Protein Size** | **GO term** | **General function (reactome^2^)** | **Specific function (ZFIN^3^)** |
| --- | --- | --- | --- | --- | --- | --- | --- | --- | --- |
| LG1:34666455-34676455 | 0 | A0A671V3S3 | Transcription cofactor vestigial-like protein 2 | VGLL2 | *Sparus aurata* (Gilthead sea bream) | 351 | GO:0005634; GO:0006355 | Gene expression | Skeletal, muscle and neural development |
| LG1:34825453-34835453 | 0 | A0A087YKW4 | LID domain-containing protein | LDB1 | *Poecilia formosa* (Amazon molly) | 388 | GO:0002040; GO:0003682; GO:0003712; GO:0005634; GO:0006357; GO:0030274; GO:0030514; GO:1900108 | Gene expression | Nervous system development. Hematopoiesis and erythropoiesis |
| LG1:35025571-35035571 | 1 | A0A671W6N0 | Bardet-Biedl syndrome 7 protein homolog | BBS7 | *Sparus aurata* (Gilthead sea bream) | 720 | GO:0005737; GO:0015031; GO:0034464; GO:0036064; GO:1905515 | Organelle biogenesis | Ciliogenesis, embryonic morphogenesis. |
| LG1:35105976-35115976 | 0 | A0A6G0I2D7 | LIM and calponin-like proteiny domains-containing protein 1 | LIMCH1 | *Larimichthys crocea* (Large yellow croaker) | 1,301 | GO:0046872 | Cell organization | Control myosin IIA, and regulate cell spreading and migration |
| LG1:35557079-35567079 | 0 | A0A669D463 | Fibroblast growth factor | FGF18a | *Oreochromis niloticus* (Nile tilapia) | 216 | GO:0007165; GO:0008083 | Growth factor | Embryonic development, cell growth, morphogenesis, tissue repair |
| LG1:35595269-35605269 | 1 | A0A671W798 | Nucleophosmin | NPM3 | *Sparus aurata* (Gilthead sea bream) | 380 | GO:0003676; GO:0005634; GO:0007165; GO:0008083 | Growth factor activator | Muscle development, Transcription |
| LG4:16465935-16475935 | 1 | A0A671Z091 | Transporter | SLC6A4 | *Sparus aurata* (Gilthead sea bream) | 575 | GO:0015293; GO:0016021 | Transport of small molecules | Dopamine metabolism and transport |
| LG4:20432926-20442926 | 1 | A0A3Q1FR53 | Phospholipid-transporting ATPase | ATP9B | *Acanthochromis polyacanthus* (spiny chromis) | 1,093 | GO:0000287; GO:0005524; GO:0015914; GO:0016021; GO:0140326 | Transport of small molecules | Endocytosis, phospholipid translocation |
| LG6:9658968-9668968 | 0 | A0A671YXI2 | Protein kinase, AMP-activated, gamma 2 non-catalytic subunit b | PRKAG2 | *Sparus aurata* (Gilthead sea bream) | 510 | GO:0032559; GO:0071900 | Metabolism | Biosynthesis of fatty acid and cholesterol |
| LG6:9676422-9686422 | 0 | M4APV5 | Protein kinase, AMP-activated, gamma 2 non-catalytic subunit b | PRKAG2 | *Xiphophorus maculatus* (Southern platyfish) | 551 | GO:0032559; GO:0071900 | Metabolism | Biosynthesis of fatty acid and cholesterol |
| LG6:9756195-9766195 | 0 | A0A3Q3XLE7 | Laminin subunit alpha 3 | LAMA3 | *Mola mola* (Ocean sunfish) | 558 | GO:0005604; GO:0007155; GO:0016020 | Cell organization | Epidermal development, Regulators including keratinocyte growth factor, epidermal growth factor and insulin-like growth factor |
| LG6:9769947-9779947 | 2 | A0A671YUC3 | Laminin subunit alpha 3 | LAMA3 | *Mola mola* (Ocean sunfish) | 558 | GO:0005604; GO:0007155; GO:0016020 | Cell organization | Epidermal development, Regulators including keratinocyte growth factor, epidermal growth factor and insulin-like growth factor |
| LG6:9920425-9930425 | 0 | A0A0S7EL66 | PPUP8465 | PPUP8465 | *Poeciliopsis prolifica* (blackstripe livebearer) | 113 |  |  |  |
| LG6:10018005-10028005 | 0 | A0A671YN70 | Potassium channel tetramerization domain containing 1 | KCTD1 | *Sparus aurata* (Gilthead sea bream) | 342 | GO:0051260 | Gene expression |  |
| LG6:10144697-10154697 | 0 | A0A671V3S3 | Transcription cofactor vestigial-like protein 2 | VGLL2 | *Sparus aurata* (Gilthead sea bream) | 351 | GO:0005634; GO:0006355 | Gene expression | Skeletal, muscle and neural development |
| LG6:14425739-14435739 | 1 | A0A3B5K6M0 | Integrase catalytic domain-containing protein |  | *Takifugu rubripes* (Japanese pufferfish) | 1,007 | GO:0003676; GO:0015074 |  |  |
| LG11:34893993-34903993 | 0 | A0A671UMV2 | Non-specific serine/threonine protein kinase | LRRK1 | *Sparus aurata* (Gilthead sea bream) | 1,949 | GO:0005524;  GO:0005525; GO:0106310; GO:0106311 | Protein metabolism | Bone development |
| LG12:2825952-2835952 | 0 | E6ZHW9 | Protein TANC1 | TANC1 | *Dicentrarchus labrax* (European seabass) | 232 |  | Signal Transduction | Sensing mechanical and thermal stimuli |
| LG16:4653138-4663138 | 1 | A0A1A8H618 | Reverse transcriptase domain-containing protein |  | *Nothobranchius korthausae* | 467 | GO:0008168; GO:0016706 | Gene expression |  |
| LG16:4995227-5005227 | 0 | A0A4Z2H9E6 | Uncharacterized protein |  | *Liparis tanakae* (Tanaka's snailfish) | 173 |  |  |  |
| LG16:5067220-5077220 | 0 | A0A671VV40 | Procollagen-proline 3-dioxygenase | P3H1 | *Sparus aurata* (Gilthead sea bream) | 751 | GO:0005506; GO:0019797; GO:0032963; GO:0031418; GO:0016705; GO:0016491; GO:0046872; GO:0051213; GO:0019797; GO:0019797; GO:0019511; GO:0019511; GO:0019511; ; | Protein metabolism | Collagen biosynthesis , bone, muscle, and nervous development |
| LG16:5086986-5096986 | 1 | A0A671W7V7 | Collagen-binding protein (Serpin H1) | SERPINH1 | *Sparus aurata* (Gilthead sea bream) | 410 | GO:0004867; GO:0005518; GO:0005615; GO:0005783 | Protein metabolism | Collagen biosynthesis, bone, and fin development |
| LG16:5107755-5117755 | 1 | A0A5C6NY95 | RNA-directed DNA polymerase from mobile element jockey |  | *Takifugu flavidus* (sansaifugu) | 859 | GO:0003964; GO:0008168; GO:0016706; GO:0016829 | Gene expression |  |
| LG16:5118678-5128678 | 0 | A0A671W9U7 | ZnMc domain-containing protein | MMP18 | *Sparus aurata* (Gilthead sea bream) | 503 | GO:0004222; GO:0008270; GO:0031012 | Elastic fibres development | embryonic development and tissue remodelling |
| LG16:5156793-5166793 | 0 | A0A3P8STB3 | Matrix metallopeptidase 1 | MMP1 | *Amphiprion percula* (Orange clownfish) | 267 | GO:0004222; GO:0008270; GO:0031012 | Elastic fibres development | embryonic development and tissue remodelling |
| LG16:5167248-5177248 | 0 | A0A3P8STB3 | Matrix metallopeptidase 1 | MMP1 | *Amphiprion percula* (Orange clownfish) | 267 | GO:0004222; GO:0008270; GO:0031012 |  |  |
| LG16:5249693-5259693 | 0 | A0A671W4Z7 | Down syndrome cell adhesion molecule homolog | DSCAM | *Sparus aurata* (Gilthead sea bream) | 2,019 |  | Cell organization | Cell adhesion and migration |
| LG16:5300383-5310383 | 1 | A0A3Q3W725 | Down syndrome cell adhesion molecule homolog | DSCAM |  |  |  |  |  |
| LG16:5455476-5465476 | 0 | A0A4Z2HFS3 | Protein spinster 2 | SPNS2 | *Liparis tanakae* (Tanaka's snailfish) | 264 | GO:0016020; GO:0016021 | Metabolism | Blood vessel morphogenesis, organ development |
| LG16:6893540-6903540 | 0 | A0A3Q3WUH7 | Family with sequence similarity 120A | FAM120A | *Mola mola* (Ocean sunfish) | 661 |  | Signal Transduction | Oxidative stress response |
| LG16:8977510-8987510 | 1 | A0A671WUN3 | periostin-like | OSF2 | *Sparus aurata* (Gilthead sea bream) | 815 | GO:0007155 | Cell organization | Bone and epidermis development, cell adhesion |
| LG16:9134115-9144115 | 0 | A0A6I9PRP0 | FRAS1-related extracellular matrix protein 2 | FREM2 | *Notothenia coriiceps* (black rockcod) | 3,120 | GO:0007154; GO:0016021 | Cell organization | Epidermal development, fin morphogenesis |
| LG16:9219843-9229843 | 0 | A0A668SAX8 | Tripartite motif containing 3b | TRIM3 | *Oreochromis aureus* (Israeli tilapia) | 551 | GO:0005737; GO:0008270 | Protein metabolism | Regulate myosins |
| LG16:9413671-9423671 | 0 | A0A6I9Q5W8 | Protocadherin-16-like | PCDH18 | *Notothenia coriiceps* (black rockcod) | 1,379 | GO:0005509; GO:0016020; GO:0007156; GO:0007155; GO:0005886; GO:0005509 | Transport of small molecules | Nervous system development |
| LG16:9538671-9548671 | 0 | A0A671VMB7 | Tripartite motif-containing protein 16-like | TRIM16 | *Sparus aurata* (Gilthead sea bream) | 522 | GO:0008270; GO:0046872 | Signal Transduction | Regulate autophagic responses, cell division |
| LG16:9607609-9617609 | 0 | A0A671W939 | T-lymphoma invasion and metastasis-... | TIAM1 | *Sparus aurata* (Gilthead sea bream) | 1,496 | GO:0005085; GO:0007264; GO:0090630 | Cell organization | Regulate RAC1,cell adhesion, growth and cytoskeletal formation |
| LG16:9620373-9630373 | 0 | A0A3Q3MEZ5 | T-lymphoma invasion and metastasis-inducing protein 1-like | TIAM1 | *Mastacembelus armatus* (zig-zag eel) | 1,680 | GO:0005085; GO:0007264; GO:0090630 | Signal Transduction |  |
| LG16:9714770-9724770 | 0 | A0A4Z2H1V9 | Metabotropic glutamate receptor 5 | GRM5 | *Liparis tanakae* (Tanaka's snailfish) | 195 | GO:0004930; GO:0005886; GO:0016021 | Signal Transduction | Nervous system development |
| LG16:9833071-9843071 | 0 | A0A6G0I7L3 | Protocadherin Fat 3 FAT tumor suppressor-like protein 3 | FAT3 | *Larimichthys crocea* (Large yellow croaker) | 1,006 | GO:0005509; GO:0016021 | Nervous system development | Specific subset of neurons during development, cartilage morphogenesis |
| LG16:10630785-10640785 | 1 | A0A671WZZ9 | Hephaestin Like 1 | HEPHL1 | *Sparus aurata* (Gilthead sea bream) | 1,098 | GO:0005507; GO:0005886; GO:0006825; GO:0006826; GO:0006879; GO:0016491 | Metabolism | Neural development |
| LG16:10780417-10790417 | 1 | A0A671WRP8 | Multidrug and toxin extrusion protein | SLC47a1 | *Sparus aurata* (Gilthead sea bream) | 517 | GO:0015297; GO:0016021; GO:0042910 | Transport of small molecules |  |
| LG16:10840197-10850197 | 1 | A0A315VJ11 | Fructose-bisphosphate aldolase | ALDOB | *Gambusia affinis* | 436 | GO:0004332; GO:0006096 | Protein metabolism | Embryo development |
| LG16:10965140-10975140 | 0 | A0A671Z3J5 | Rho GTPase-activating protein 42-like | ARHGAP42 | *Sparus aurata (Gilthead sea bream)* | 852 | GO:0007165; GO:0005096; GO:0050790; | Signal Transduction |  |
| LG16:11023266-11033266 | 0 | A0A3B3CK08 | Uncharacterized protein |  |  |  |  |  |  |
| LG16:11122066-11132066 | 1 | A0A6J2R456 | cilia- and flagella-associated protein 54 | CFAP54 | *Cottoperca gobio (Frogmouth)* | 2576 | GO:0031514 | Motile cilium |  |
| LG16:19043759-19053759 | 0 | A0A671Z3J5 | Dihydropyrimidinase like 5a | DPYSL5A | *Sparus aurata* (Gilthead sea bream) | 626 | GO:0005737; GO:0007411; GO:0008017; GO:0016810 | Nervous system development | Axon guidance, neural development |
| LG16:25521748-25531748 | 0 | A0A671WIC5 | MKS transition zone complex subunit 1 | MKS1 | *Sparus aurata* (Gilthead sea bream) | 563 | GO:0005737; GO:0030030 | Cell organization | Epidermal development; organism length |
| LG16:28875624-28885624 | 0 | A0A6J2R3I5 | Guanylate cyclase activator 1d | GUCA1D | *Cottoperca gobio* (Frogmouth) | 199 | GO:0005509 | Signal Transduction | Enables calcium ion binding activity and calcium sensitive guanylate cyclase activator activity |
| LG16:28887646-28897646 | 0 | A0A671V6T3 | Alpha-1,3-glucosyltransferase | ALG8 | *Sparus aurata* (Gilthead sea bream) | 506 | GO:0005789; GO:0006486; GO:0006490; GO:0016021; GO:0042283 | Protein metabolism |  |
| LG16:28887646-28897646 | 0 | A0A671V0B0 | Coiled-coil domain containing 90B | CCDC90B | *Sparus aurata* (Gilthead sea bream) | 238 | GO:0016020; GO:0016021; GO:0005739 | Signal Transduction | Bone and cartilagous development. Brain development |
| LG16:30122546-30132546 | 0 | XM_37101526 | Growth differentiation factor 5 | GDF5 | *Acanthopagrus latus* | 1201 | GO:0005104; GO:0009887; GO:0008284; GO:0008543; GO:0005615; GO:0010628; GO:0005737; GO:0001934; GO:0030154; GO:0008083; GO:0030334; GO:0008083; GO:0005576; GO:0008543; GO:0005104; GO:0008083; GO:0005576; GO:0051781; GO:0061550; GO:0005576 | Growth factor | Regulates the development of numerous tissue and cell types, include bone, cartilage, fat, muscle, and neuron formation |
| LG16:30167878-30177878 | 0 | A0A4U5V666 | Transmembrane protein 132E | TMEM132E | *Collichthys lucidus* (Big head croaker) | 373 |  | Protein metabolism | Regulation of insulin-like growth factor, involved in cell development, nervous system |
| LG16:30261705-30271705 | 0 | A0A671VPR0 | Solute carrier family 25 member 14 | SLC25A14 | *Sparus aurata* (Gilthead sea bream) | 298 | GO:0005743; GO:0006839; GO:0016021; GO:0055085 | Metabolism | Mitochondrial |
| LG17:8089-18089 | 0 | A0A6P7NG69 | KIAA0930 homolog | kiaa0930 | *Betta splendens* (Siamese fighting fish) | 538 | GO:0016020; GO:0016021 | Signal Transduction | Homeostasis |
| LG18:31180773-31190773 | 0 | A0A671TTN2 | Uncharacterized protein |  | *Sparus aurata* (Gilthead sea bream) | 142 |  |  |  |
| LG22:2458072-2468072 | 0 | A0A5C6PF31 | Uncharacterized protein |  | *Takifugu flavidus* (sansaifugu) | 170 |  | Gene expression | DNA replication, chromatid cohesion |
| LG22:9897982-9907982 | 0 | A0A0F8C4M4 | Wings apart-like protein | WAPLB | *Larimichthys crocea* (Large yellow croaker) | 1,171 |  |  |  |
| LG23:8316088-8326088 | 0 | A0A671W8M8 | Myb/SANT-like DNA-binding domain-containing protein 1 | MSANTD1 | *Sparus aurata* (Gilthead sea bream) | 279 | GO:0003677 | Signal Transduction | DNA binding implicate in ciliogenesis |
| 1: https://www.uniprot.org/ 2: https://reactome.org/ 3: https://zfin.org/ |  |  |  |  |  |  |  |  |  |


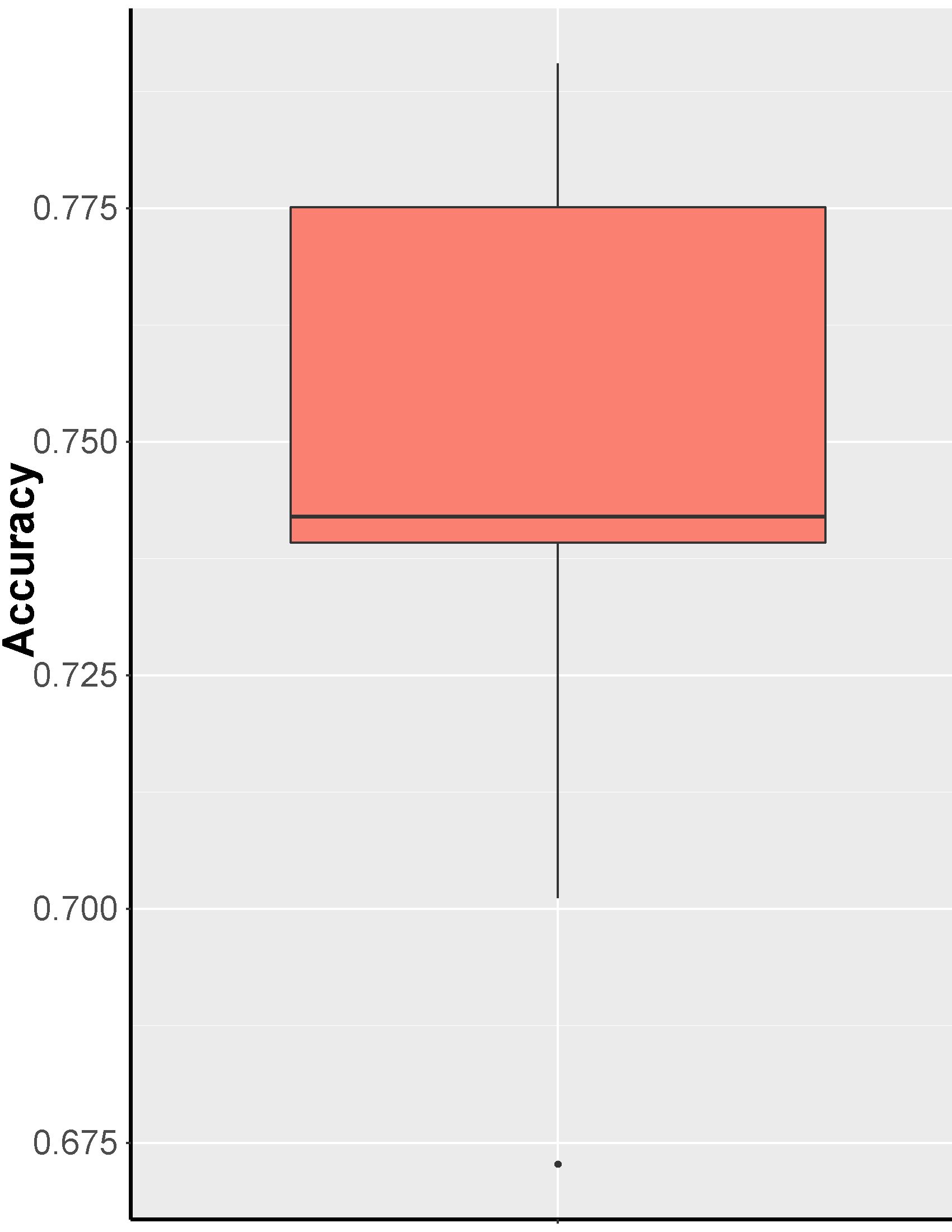


**Figure S1** Boxplot showing the mean (0.744) and distribution of the accuracy of phenotype (small or large) prediction base on bayesR results using 17,490 SNPs for 363 Australian snapper (*Chrysophrys auratus*). The plot is based on 10 replicates with randomly selected 80% of the samples as training data and 20% of the samples as validation data.
